# Novel Bluetongue Virus Serotype from Kuwait

**DOI:** 10.3201/eid1705.101742

**Published:** 2011-05

**Authors:** Sushila Maan, Narender S. Maan, Kyriaki Nomikou, Carrie Batten, Frank Antony, Manjunatha N. Belaganahalli, Attia Mohamed Samy, Ammar Abdel Reda, Sana Ahmed Al-Rashid, Maha El Batel, Chris A.L. Oura, Peter P.C. Mertens

**Affiliations:** Author affiliations: Institute for Animal Health, Woking, UK (S. Maan, N.S. Maan, K. Nomikou, C. Batten, F. Antony, M.N. Belaganahalli, C.A.L. Oura, P.P.C. Mertens);; Public Authority of Agriculture Affairs and Fish Resources, Kuwait City, Kuwait (A.M. Samy, A.A. Reda, S.A. Al-Rashid, M. El Batel);; Cairo University, Cairo, Egypt (A.M. Samy)

**Keywords:** viruses, Bluetongue virus, serotypes, Kuwait, phylogenetic analysis, Seg-2, Seg-3, dispatch

## Abstract

Sheep and goats sampled in Kuwait during February 2010 were seropositive for bluetongue virus (BTV). BTV isolate KUW2010/02, from 1 of only 2 sheep that also tested positive for BTV by real-time reverse transcription–PCR, caused mild clinical signs in sheep. Nucleotide sequencing identified KUW2010/02 as a novel BTV serotype.

Bluetongue virus (BTV) infects ruminants, camelids, and occasionally large carnivores. Clinical signs of bluetongue disease (BT) are usually more severe in sheep or white-tailed deer, particularly in populations previously unexposed to the virus; cattle and goats are often asymptomatic (*1*). Initial diagnosis of BT based on clinical signs can be confirmed by virus isolation and characterization or identification of viral RNA by reverse transcription PCR.

BTV particles contain 3 concentric protein layers surrounding 10 linear double-stranded RNA genome segments, identified as segment-1 to segment-10 (Seg-1 to Seg-10) in order of decreasing size (from 3,954 bp to 822 bp) (*2*). Twenty-five BTV serotypes have been identified on the basis of the specificity of reactions with neutralizing antibodies generated by their mammalian hosts (*3*). Consequently, BTV outer capsid proteins, particularly viral protein (VP) 2 (encoded by Seg-2), show sequence variations that determine virus serotype (*4*). Other BTV proteins, including subcore shell protein VP3(T2) encoded by Seg-3, are more highly conserved (*2*). Phylogenetic comparisons of Seg-3 sequences have been used to identify different BTV topotypes and distinguish different *Orbivirus* species (*4*).

BTV has been reported in several Middle Eastern countries (Egypt, Jordan, Syria, Turkey, Cyprus, and Iraq) since 1951 (*5*). In 2008, Egypt reported the absence of BT, and Egypt is the only country in the region to have prohibited BTV vaccination (*5*). Iran reported outbreaks of BT in 2008, and Saudi Arabia reported infection without clinical signs, although the serotype(s) were not identified (*5*). Multiple serotypes were detected in Israel during 2008 (*5*) and Oman in 2009 (S. Maan et al., unpub. data). We report characterization of a novel BTV serotype identified in Kuwait in 2010.

## The Study

During February 2010, sheep and goats in the Abdali region of Kuwait, close to the Iraq border, showed the following clinical signs consistent with BT: lameness, coughing, mouth lesions, stillbirth, congenital abnormalities, pneumonia, enteritis, and hepatitis. Forty-six of 48 serum samples were positive for BTV-specific antibodies by competitive ELISA (Investcare-Vet, London, UK) at the Veterinary Diagnostic Laboratory and Animal Research Center in Kuwait, or by double antigen-recognition ELISA (ID Vet, Montpellier, France) at the Institute for Animal Health in the United Kingdom.

Twenty-six EDTA-treated blood samples, 4 spleens, and 1 liver sent for analysis for BTV to the World Organisation for Animal Health reference laboratory at the Institute for Animal Health (Woking, UK) all gave negative results by real-time reverse transcription–PCR (rRT-PCR) targeting either BTV Seg-1 (*6*) or Seg-1 and Seg-5 (*7*). However, 2 blood samples (from animals 364 and 374) were positive in assays selective for Seg-10 (*8*), with cycle threshold (C_t_) values of 35 and 28, respectively. Previous attempts to isolate BTV from blood samples with C_t_ values >32 were usually unsuccessful, and no further work was done with animal 364. Washed blood from animal 374 (reference collection sample KUW2010/01) (*9*) was injected into embryonated chicken eggs. Although no hemorrhages were detected, the virus was passaged twice in BHK-21 cells (isolate KUW2010/02), producing atypical cytopathic effects.

**Figure 1:**
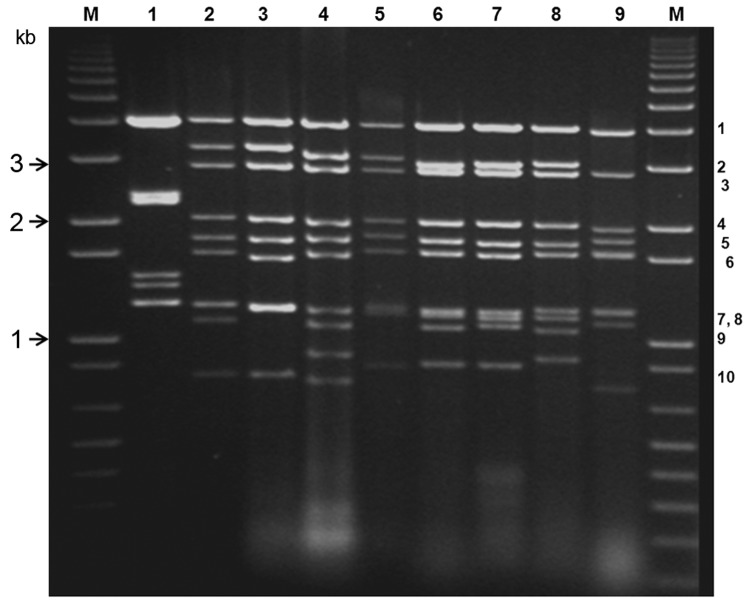
Electrophoretic analysis of genomic double-stranded RNAs from the *Orbivirus* species and mammalian orthoreoviruses*.* Bluetongue virus double stranded RNA preparations were analyzed by electrophoresis in a 1% agarose gel containing 0.5 μg/mL ethidium bromide and visualized by exposure to ultraviolet light. Genome segments are numbered, in order of decreasing molecular weight. DNA markers were run (lanes M) to enable estimation of molecular weights. Lane 1, orthoreovirus (MOR2004/01); 2, equine encephalosis virus (EEV-1/RSA1976/03); 3, African horse sickness virus (AHSV-1/RSArrah/01); 4, Palyam virus (PALV-SUD1982/03); 5, epizootic hemorrhagic disease virus (EHDV-4/ NIG1968/01); 6, bluetongue virus (BTV-15/RSArrrr/15); 7, bluetongue virus (BTV-26/KUW2010/02); 8, Tilligerry virus (TILV-AUS1978/03); 9, Chobar Gorge virus (CGV).

KUW2010/02 RNA analyzed by agarose gel electrophoresis generated a genome segment migration pattern (electropherotype) typical of BTV (Figure 1). Although KUW2010/02 was negative by BTV-specific rRT-PCR selective for Seg-1 (*6*), it had C_t_ values of 16.8 in Seg-10–specific assays (*8*). Identification of KUW2010/02 as BTV was confirmed by indirect antigen-sandwich ELISA selective for core protein VP7(T13) (*10*) with optical density at 490 nm values >0.15 (equivalent BTV titer of 6.75 log_10_ 50% tissue culture infective dose/mL).

RNA from KUW2010/01 and KUW2010/02, extracted by using TRIzol (Invitrogen, Carlsbad, CA, USA) (*11*), gave uniformly negative results by type-specific rRT-PCRs targeting Seg-2 of BTV serotypes 1–25 (kits supplied by Laboratoire Service International, Lissieu, France). However, full-length cDNA copies of Seg-2 (2,929 bp) and Seg-3 (2,773 bp) from KUW2010/02 were synthesized and then sequenced as described (*11*), showing conserved terminal regions typical of BTV (5′-GUUAAA...........ACUUAC-3′) (*2*). BLAST (*www.ncbi.nlm.nih.gov/BLAST)* analysis of KUW2010/02 Seg-2 and Seg-3 sequences (GenBank accession nos. HM590642 and HM590643) showed highest identity with equivalent genome segments of other BTVs, although for Seg-2 the search algorithm was changed from megablast (highly similar sequences) to blastn (somewhat similar sequences).

Phylogenetic analysis of KUW2010/02 Seg-3/VP3(T2), conducted by using neighbor-joining methods and p-distance matrices (*12*), showed nucleotide/amino acid identity levels of 73.7%/87.6% to 76.6% /88.9% with other BTVs. Seg-3 of none of the previously characterized BTVs showed close relationships to KUW2010/02; BTV-1/GRE2001/05 and BTV-25/TOV were most closely related (GenBank accession nos. DQ186822 and GQ982523, respectively), which indicates that KUW2010/02 represents a distinct geographic cluster or topotype (*4*).

**Figure 2:**
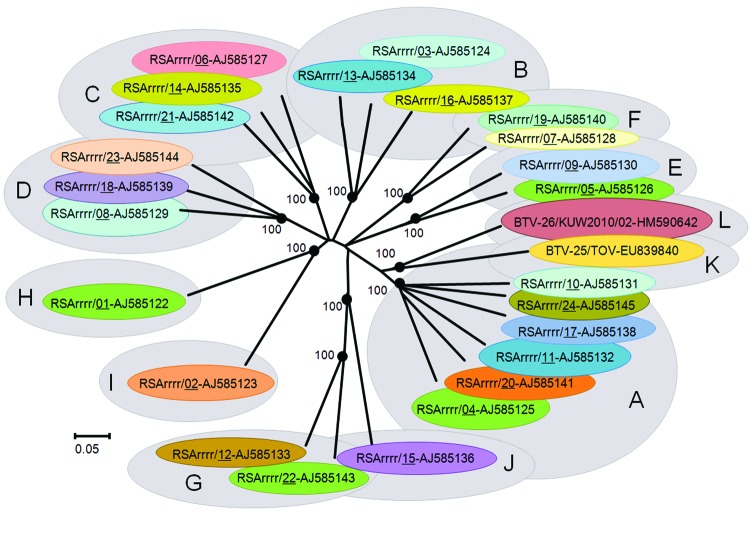
Neighbor-joining tree showing relationships between genome segment-2 (Seg-2) from KUW2010/02 with the 25 reference strains of different bluetongue virus (BTV) serotypes. The tree was constructed by using distance matrices, generated by using the p-distance determination algorithm in MEGA 4.1 (500 bootstrap replicates) (*12*). The 10 evolutionary branching points are indicated by black dots on the tree (along with their bootstrap values), which correlate with the 11 Seg-2 nucleotypes designated A–L. BTV-26 (KUW2010/02),forms a new 12th Seg-2 nucleotype (L). Members of the same Seg-2 nucleotype are characterized by 66.9% identity in their Seg-2 nucleotide sequences; members of different nucleotypes show 61.4% identity in Seg-2 (*4*). The scale bar indicates the number of substitutions per site. The tree based on the amino acid sequences of viral protein 2 showed very similar topology. GenBank accession numbers of Seg-2 used for comparative analyses: AJ585122–AJ585145, EU839840.

Seg-2/VP2 of KUW2010/02 showed nucleotide/amino acid identity levels of 42.8%/28.3% to 63.9%/61.5% with previously recognized BTV serotypes and was most closely related to BTV-25 (nucleotype K, Figure 2). Reference strains of BTV-10 and BTV-17 from the United States (nucleotype A) were the next most closely related, with 61.8%/58.1% and 62%/57.7% nt/aa identity, respectively. This places KUW2010/02 as a distinct virus type within a novel 12th Seg-2 nucleotype L (*4*; Figure 2). ClustalX (*www.clustal.org)* alignments and neighbor-joining trees also confirmed the identity of KUW2010/02 as a BTV. Virus neutralization tests of KUW2010/02 (*13*) that used reference guinea pig immune serum against BTV types 1–24 and antiserum from goats previously infected with BTV-25 showed no reduction in KUW2010/02 infectivity (data not shown), demonstrating that it does not belong to BTV serotypes 1–25.

## Conclusions

Most serum samples tested from sheep and goats in Kuwait showing clinical signs of disease were seropositive for BTV-specific antibodies. However, BTV RNA was detected in only 2 sheep serum samples (animals 364 and 374), suggesting that the clinical signs were not caused by ongoing BTV infection. All samples were also tested for peste des petits ruminants virus by rRT-PCR (*14*), but results were uniformly negative.

BTV RNA was detected by using a BTV Seg-10–specific rRT-PCR (*8*) previously used to detect BTV-25 in Switzerland (*15*). However, BTV Seg-1–specific or Seg-1– and Seg-5–specific assays (*6**,**7*) failed to detect KUW2010/02, identifying it is an unusual or atypical BTV strain. Alignment of the Seg-1–specific and Seg-5–specific primers and probes with KUW2010/02 sequences identified numerous mismatches that would prevent detection of the viral RNA (data not shown). However, the probe and primers designed by Orrù et al. (*8*) showed a perfect match with Seg-10 of KUW2010/02, demonstrating the need for appropriate rRT-PCR protocols to detect this virus. Agarose gel electrophoresis analysis of KUW2010/02 genome segments generated a migration pattern typical of BTV (Figure 1). Levels of nucleotide/amino acid identity of Seg-3 (up to 76.6%/89%) of KUW2010/02 with other BTV isolates also identified it as BTV.

At peak viremia, a previously unexposed sheep infected with a virulent BTV strain could be expected to show C_t_ values ≈20. The C_t_ of 28 obtained with blood of animal 374 (KUW2010/01) indicates a low viremia, suggesting that the severe clinical signs observed were not caused by BTV. Experimental infection of previously unexposed sheep with KUW2010/02 also caused only mild clinical signs (data not shown).

Different isolates of a single BTV serotype show >68.4%/72.6% nt/aa identity in Seg-2/VP2, with 40.5%/22.1% to 71.5%/77.8% identity between different serotypes (*4*). KUW2010/02 showed only 42.8%/28.3% to 63.9%/61.5% identity in Seg-2/VP2 with recognized BTV serotypes, consistent with membership of a novel 26th BTV type and Seg-2 nucleotype (L) (*4*). These conclusions were supported by virus neutralization test results. The sequence data presented here will help support development of new diagnostic tools (RT-PCR–based typing assays) to determine the incidence and distribution of this novel serotype, as well as natural reservoir(s) and insect vectors.
